# Siloranes–Suitability of a Novel Adhesive for Orthodontic Bracket Bonding

**DOI:** 10.3390/dj9110135

**Published:** 2021-11-17

**Authors:** Lorenz Brauchli, Markus Steineck

**Affiliations:** Clinic of Orthodontics, School of Dentistry, University of Basel, 4051 Basel, Switzerland; markus.steineck@unibas.ch

**Keywords:** silorane, shear bond strength, enamel, bracket bonding

## Abstract

Recently, an epoxy-based resin-Filtek Silorane-has been proposed for restorative fillings. It was the aim of the investigation to evaluate the suitability of this novel resin for orthodontic bracket bonding on unground enamel. Shear bond strength was measured for two adhesives-Filtek Silorane, Transbond XT-in combination with steel, ceramic and polymer brackets. For Filtek Silorane etching was performed with the Silorane self-etching primer, as well as phosphoric acid. The Transbond XT samples were etched with phosphoric acid only and served as the control group. All samples were thermo-cycled (1000×, 5–55 °C). Shear testing was carried out with an Instron 3344. In addition, ARI scores were evaluated. The Shear bond strength showed a weak adhesion of Filtek Silorane to unprepared enamel, either with the self-etching primer or the conventional etching (0.87–4.28 MPa). The Shear bond strength of the control group was significantly higher (7.6–16.5 MPa). The ARI scores showed a clear failure at the enamel-adhesive interface for all Filtek Silorane samples. For the combination of Transbond XT and different brackets the failure was found at the adhesive–bracket interface. The novel epoxy-based resin Filtek Silorane is not appropriate for bracket bonding to unprepared enamel.

## 1. Introduction

Since the introduction of dental adhesives by Buonocore [1], many modifications and attempts to develop resins which were more stable, aesthetically pleasing, or had a simplified handling were undertaken [2]. The polymerization chemistry of the vast majority of resins is that of a methacrylate resin. These resins polymerize by the opening of double bindings through free radicals, which thereafter can interact with opened bindings of other methacrylate molecules. One of the major problems with methacrylate- based resins was the relatively high shrinkage during polymerization. The shrinkage is due to an increase in viscosity during polymerization which inhibits the flow of unpolymerized methacrylates and radicals in the post-gel state and in turn leads to a contraction of the material [3]. This shrinkage is of major concern for the marginal integrity of large restorations. Over the last decade, the percentage of shrinkage in current methacrylate resins for posterior restorations has dropped to 1–3%. This low shrinkage was achieved by two modifications of the original resin. The prolongation of the methacrylate chains from 86.1 g/mole to 514.6 g/mole has reduced the shrinkage from 22% to 8% [4]. In addition, the increase in fillers, which has become possible by adding nano particles to the resins, allowed for the reduction to the current shrinkage of 1–3% [4]. Still, shrinkage remains one of the major concerns in restorative dentistry, which is also documented by the vast number of studies addressing the topic.

Another way to reduce shrinking was followed by researchers who searched for alternative resins to methacrylates. Epoxy based resins have a fundamentally different polymerization. The polymerization is induced by cations which lead to an opening of the ring structure of the epoxides. The polymerization of these oxyrane rings is accompanied by a far lesser degree of shrinkage [4,5], or even a slight expansion [6]. Subsequently the marginal integrity and micro-leakage at the dentine resin interface were reduced in comparison with conventional composites [7]. However, other problems had to be addressed with the use of epoxy-based resins, such as, for example, their cytotoxicity [5]. It is known that epoxids can induce chromosomal aberrations [5]. However, by combining the oxyrans with strongly hydrophobic siloxanes, which are oxidized silicones, no cytotoxicity could be found for the novel resin [5]. These composites were called Siloranes and were described by Guggenberger and Weinmann and patented by 3M/ESPE [8]. The inhibition of the hydrolysis of the oxyranes by the hydrophobic siloxanes [9] as well as the low cytotoxicity of the monomers [10] are discussed as reasons for the high biocompatibility of siloranes. It is also suggested that the hydrophobity of the siloranes is responsible for the lower bacterial affinity [11] and the lesser absorption of dyes reported in the literature [4]. The mechanical stability is reported to be equal to that of methacrylates in respect to e-modulus and flexural strength [11]. Marginal adaption and micropermeability are controversially discussed in the existing literature, suggesting no advantage for siloranes [12,13]. Shear bond strengths tend to be lower, both to dentin [14] and enamel [15], as well as to aged methacrylate blocks simulating the repair of older restorations [16].

For orthodontic bonding, many of the factors mentioned above may not be of utmost importance. Shrinkage is probably of little concern, as only a very thin composite layer is formed in between bracket and enamel. Additionally, the absorption of water and degradation of the resin is probably of lesser concern as the bond is only expected to last for 1–3 years. The lesser absorption of dyes is certainly an advantageous feature, as well as the lower adhesion of bacteria to the hydrophobic silorane surface. The major concern in orthodontic bonding however is the shear bond strength to unetched enamel, which has not been addressed to our knowledge. A study by the developers suggested a good adhesion to dentine as well as ground enamel [17,18]. The hypothesis of the present study was the following. If Filtek Silorane has a strong adhesion to unground enamel, as well as different bracket materials, then this novel adhesive system will show shear bond strengths similar or higher than the conventional methacrylate systems and can therefore be recommended for orthodontic bracket bonding.

## 2. Materials and Methods

Two bonding systems were evaluated for their shear bond strength to unground enamel and three different bracket types. The adhesives tested were Transbond XT (3M/ESPE, Monrovia, CA, USA), which is a standard adhesive in orthodontic bonding and served as a control group and Filtek Silorane (3M/ESPE, Monrovia, CA, USA).

Both adhesives were tested with a ceramic bracket (Clarity, 3M/ESPE, Monrovia, CA, USA), a metal bracket (Victory, 3M/ESPE, Monrovia, CA, USA) and a composite bracket (Esthetic Line, Forestadent, Pforzheim, Germany). Freshly extracted bovine teeth were used as human enamel substitute. All teeth were extracted, the roots shortened, and the pulp extirpated within 24 h. Thereafter, they were stored at room temperature in frequently changed tap water for 1–3 days. The crowns were thoroughly pumiced before etching. A contra angle handpiece with a brush and unfluorized pumice (Kerr Pumice Fine, Orange, CA, USA) was used at a revolution of 2500 rpm for 10 s per tooth.

A total of 135 samples were distributed to 9 groups. All groups of 15 samples for Transbond XT were etched with 35% phosphoric acid for 30 s and primed with Transbond MIP (3M/ESPE, Monrovia, CA, USA). The Transbond MIP was light cured for 10 s. For Filtek Silorane, two etching modalities were used. All groups were etched with the Silorane self-etching primer. However, three groups of 15 samples antecedently were etched with conventional 35% phosphoric acid, rinsed, and dried with air. The contact time for conventional etching was 30 s and for the Silorane self-etching primer 15 s. After etching the teeth with the Silorane self-etching primer they were dried with a 10 s blow of compressed air and light cured for 10 s. Silorane bond was applied, dried with a blow of air of 10 s, and light cured for another 10 s. Finally, the brackets were placed on the teeth with the respective adhesive, excess bonding material was carefully removed, and the samples were light cured for 20 s with an Ortholux LED curing light (3M/ESPE, Monrovia, CA, USA).

The bonding was followed by 1000 thermo-cycles between 5 °C (±1 °C) and 55 °C (±1 °C) at a rate of 60 s per cycle and a time of submersion in each water bath of 25 s. After the thermo-cycles the roots of the teeth were embedded in a methacrylate resin (Technovit 4071, Heraeus Kulzer, Wehrheim, Germany). Thereby, attention was paid to align the bonding surface parallel to the prospective force vector of the shear mechanism. The shear-dye had a distance to the tooth surface of 0.5 mm. Shear bond strength was measured with an Instron 3344 (Instron Corp., Wilmington, DE, USA) at a crosshead speed of 1 mm/min. In addition, the ARI (adhesive remnant index) scores according to Artun and Bergland [19] were evaluated for all samples by estimating the amount of bonding material remaining on the two surfaces under 3.5-fold magnification. A score of 0 indicated samples with no adhesive left on the enamel, one with less than 50% on the enamel, two with more than 50% on the enamel and a score of three with all adhesive remaining on the tooth.

GraphPad Prism (GraphPad, San Diego, CA, USA) was used for the statistical analysis. As not all groups showed a normal distribution according to Kolmogorov–Smirnov, significant differences were calculated at a level of *p* ≤ 0.05 using an ANOVA with Kruskal–Wallis and Dunn’s post-test.

## 3. Results

**Figure 1:**
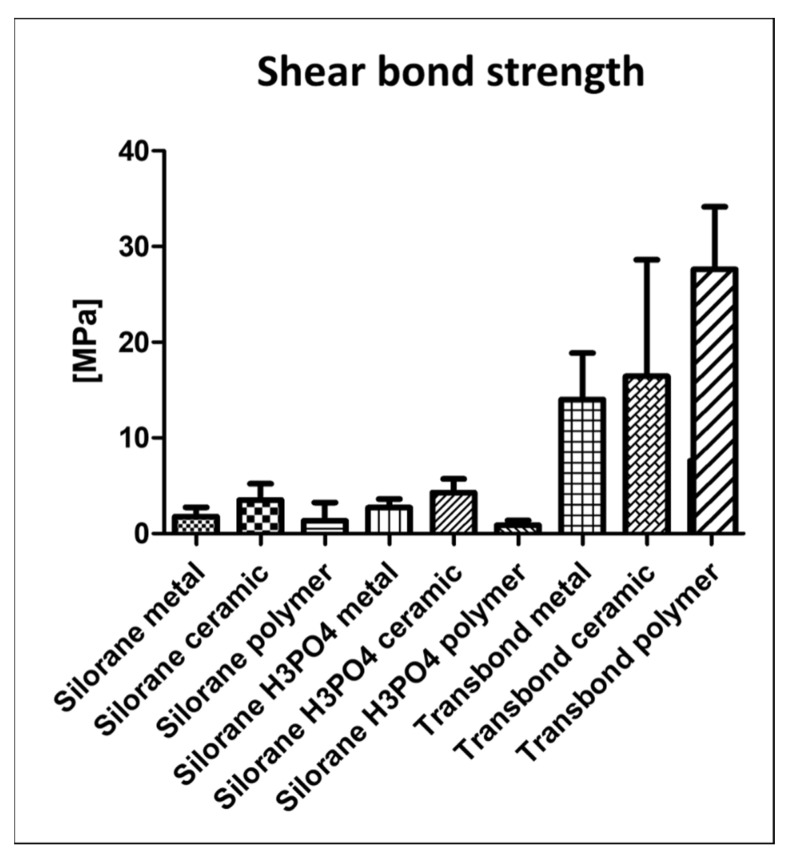
Mean shear bond strength and standard deviations. Significant differences (*p* ≤ 0.05) were found between the Filtek Silorane and the Transbond XT groups.

**Figure 2:**
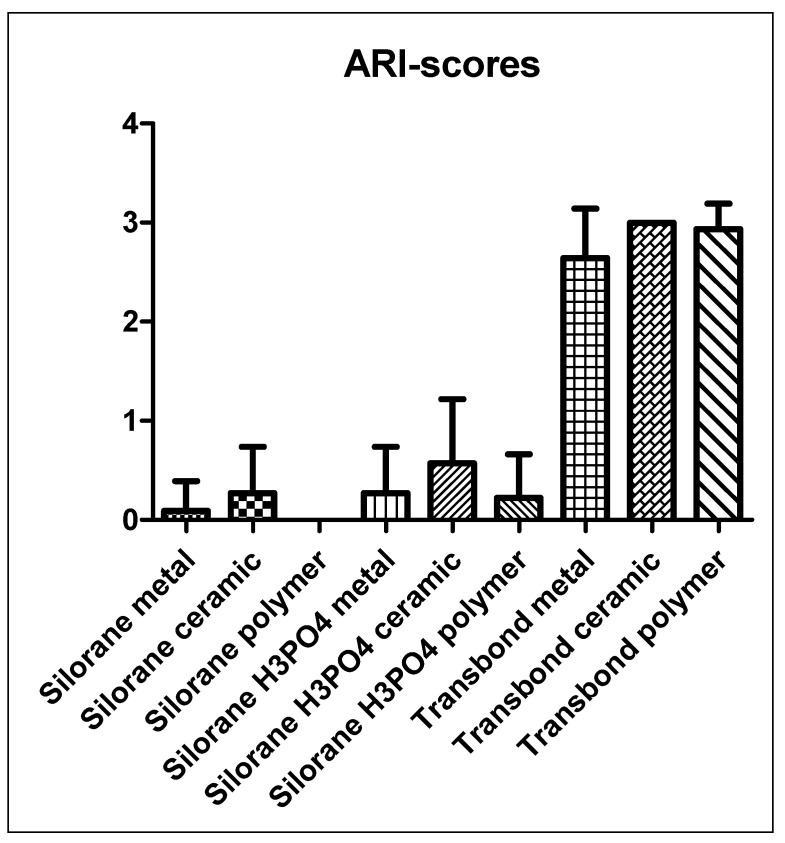
ARI scores of all test groups. High ARI scores indicate a fracture at the adhesive–enamel interface. Low scores indicate fractures between adhesive and bracket.

Thermo-cycling led to the spontaneous detachment of 23% of all Filtek Silorane samples, whether etched with phosphoric acid or not prior to using the Silorane self-etching primer. No samples were lost through thermo-cycling in the Transbond XT groups. The Silorane samples, either treated with conventional etching in addition to self-etching or self-etching alone, showed very low bonding forces to the untreated bovine enamel and were significantly lower (*p* ≤ 0.05) than all Transbond XT groups (Table 1, Figure 1). The ARI scores showed a consistent detachment at the enamel-adhesive interface for all Silorane groups, whereas the Transbond XT groups showed a fracture at the bracket-adhesive interface (Table 1, Figure 2).

## 4. Discussion

As extracted human teeth are ever more difficult to obtain due to advances in conservative dentistry, bovine incisors were used in the present study as a substitute for human enamel. The use of bovine enamel instead of human enamel has been recommended by the ISO 11,405 norm for adhesive shear testing and is well documented in literature where similar [20,21,22,23] or slightly reduced [24] bond strengths were found. The retentive etching pattern was found to differ only slightly between the two species with no effect on bond strength [22]. Histochemical, as well as anatomical, observations for both species were found to be essentially similar [22,23,25]. The use of bovine instead of human enamel seems, therefore, to be justified.

Optimal shear bond strength for orthodontic bonding should allow for little bracket failures during treatment as well as easy debonding at the end of treatment to avoid hypersensitivity due to bracket removal [26]. The shear bond strength measured for the adhesion of Filtek Silorane to unground bovine enamel was lower than the minimum forces recommended by Reynolds [27]. This was true for both etching techniques investigated. The results obtained in the present study were much lower than the shear bond strength reported by the developers of the Silorane technology [17,18]. However, there are two important differences in the experimental setup of the initial reports and the present investigation. In the initial studies, the bovine incisors were ground to expose enamel or dentine. While this procedure mimics the tooth surface present after preparation of a cavity, it does not represent the orthodontic conditions for the investigation of shear bond strength. It is known that the outer surface of enamel is more mineralized, and adhesion may be reduced compared to ground enamel [28,29]. However, the large difference in bond strength cannot be explained. The second difference in the research protocol between the two initial research protocols and this study was the lack of any thermo-cycling in the former investigations. It is known that thermo-cycling can strongly influence bond strength [30,31,32,33]. The difference in thermal expansion between enamel and composite causes stress in the adhesive interface. One possible explanation for the lower shear bond strength found for the Silorane samples might be a higher susceptibility to thermal stresses. The commonly used thermo-cycles between 5 °C and 55 °C might not be representative of an in vivo situation. However, the bond strength of the methacrylate resin Transbond XT to enamel was not strongly influenced by the thermo-cycles, neither in the present investigation nor in the literature [32,34,35]. In an earlier investigation [36] of the bond strengths of seven self-etching primers, spontaneous detachments of some self-etching primers were found under the same thermo-cycles where there has been no effect of thermo-cycles on conventional etching. The thermo-cycles seem to be highly discriminative in regard to systems which are stress tolerant and those which are not.

The ARI scores for Transbond XT samples in combination with all brackets mostly showed a detachment at the bracket–adhesive interface. As the ARI scores for Filtek Silorane in combination with all brackets were very low, thus indicating a failure at the enamel-adhesive interface, the bonding properties of Filtek Silorane to the different bracket materials tested could not be further evaluated. In conclusion the bond strength of Filtek Silorane was insufficient for bonding to unprepared enamel.

## 5. Conclusions

At the present stage of development, siloranes are not an alternative to conventional methacrylate adhesives in orthodontic bonding.

## Figures and Tables

**Table 1:** Comparison of the mean shear bond strength [N], standard deviations (SD) and ARI. The column significance shows differences according to the shear bond strength (*p* ≤ 0.05).

Adhesive and Etching Mode	Group Name, Bracket Type, n	Shear Bond Strength [MPa], (SD)	Significance at *p* ≤ 0.05	ARI Score
Filtek Silorane, Silorane self-etching primer	A, ceramic, 14	3.50 (1.71)	G–K	0.27
	B, polymer, 4	1.37 (1.87)	G–K	0
	C, metal,11	1.77 (0.97)	G–K	0.09
Filtek Silorane, conventional etching, Silorane primer	D, ceramic, 15	4.28 (1.43)	G–K	0.57
	E, polymer, 9	0.87 (0.53)	G–K	0.22
	F, metal, 15	2.71 (0.91)	G–K	0.27
Transbond XT, Conventional etching, Transbond MIP	G, ceramic, 15	16.5 (12.2)	A–F, K	3.0
	H, polymer, 15	7.62 (3.62)	A–F, K	2.9
	I, metal, 15	14.0 (4.89)	A–F, K	2.6

## Data Availability

Department of Orthodontics, University of Basel, Switzerland.
